# A metric of the difference between perception of security and victimisation rates

**DOI:** 10.1186/s40163-016-0060-y

**Published:** 2016-10-28

**Authors:** Rafael Prieto Curiel, Steven Richard Bishop

**Affiliations:** University College London, Gower Street, London, WC1E 6BT UK

**Keywords:** Security, Perception, Ranking, Correlation, Kendall’s tau coefficient, Mexico

## Abstract

A person’s perception of the level of security at a specific location depends on many factors, including past experiences in that location, the actual crime suffered by the population and more. Thus, when the individual perception that a location is insecure becomes the general rule is when the perception of security becomes an attribute of the region rather than the fears of some of its individuals, hence the relevance of aggregating individual perceptions of security into a single regional perception of security. Residents of two different regions, which have the same levels of crime, of a similar nature, may have different perceptions of the level of security. The perception of security associated with a particular place is relevant by itself but is much more useful when compared to the perception of other regions or when the perception changes over time and hence a ranking of the perception levels from different places would be a useful tool. A metric is suggested here to determine first the regional perception of security from a location and then to quantify its relationship with different victimisation rates. We quantify the relationship between the perception of security and different victimisation rates, based on data obtained from Mexico through victimisation surveys.

## Background

The perception of security or insecurity might cause people to change their behaviour, for instance, by avoiding unnecessary risk when commuting back home Jackson and Gray (2010). Today, in our cities and countryside, the perception of security has become a serious issue, perhaps particularly for urban populations Carro et al. (2010). If the perception of security in a particular region is deemed to be out of line with actual victimisation rates, then this raises the question of what our policy makers can do to alter this perception to create vibrant, and economically successful regions. Falsely based perceptions affect the efficiency of the security systems, since governments are encouraged to spend resources, such as an increased number of police officers, or even introduce urban interventions, in places where people are more concerned, but not necessarily where action is most needed or where it could have the greatest impact Grogger and Weatherford (1995).

In a particular region, people’s perception of the level of crime is formed by a number of factors where the first and most clear one is whether they were personally the actual victims of a crime: past victimisation almost doubles the odds ratio of a person having fear of crime Tseloni (2007). However, past victimisation is not the only factor that contributes to a person fearing crime or perceiving a region as insecure and one of the main reasons is that, fortunately, crime is a rare event. For example, the International Crime Victims Survey shows that less than 3% of the population from the surveyed countries experiences a theft from person during the period of 1 year Tseloni et al. (2010), and rates are similarly low for other types of crime. Hence, more frequently a person experiences indirect victimisation via interactions with friends, neighbours or through media rather than experiencing actual crime Gilchrist et al. (1998).

There are many other reasons which affect whether a person feels secure in terms of crime or not. It was shown, for example, that some area characteristics, such as the economic level or the amount of people of age between 16 and 24, might act as a better predictor of fear of crime, even better than the actual crime rates Kershaw and Tseloni (2005). Another relevant factor to consider is the role that media plays in the fear of crime and perhaps we should also now include social media. It is estimated Chadee and Ditton (2005) that less than 1% of the crime features in the newspapers and that crimes of a sexual or a violent nature have a much higher probability to appear on the news Ditton and Duffy (1983). In fact, even though murder is one of the least frequent crimes, it makes up of nearly one-third of the crime stories in the newspapers Liska and Baccaglini (1990). Thus, crimes with a sexual or violent component might be perceived as being much more frequent than they actually are and hence perhaps feared more than they actually should be.

The perception of security and the fear of crime have been studied from many angles: from its social and psychological construction Farrall et al. (2000); its relation with the environment Brunton-Smith and Sturgis (2011); the impact of past victimisation to the current perception Hale et al. (1994); to more methodological aspects such as how the fear of crime is measured Farrall et al. (1997). In order to build models, one of the most common tools to understand the interactions are victimisation oriented surveys, such as the Crime Survey for England and Wales Office for National Statistics (2016) or the National Crime Victimization Survey (NCVS) in the United States Bureau of Justice Statistics (2016), which allow agencies and governments to obtain precise information about the fear of crime. These surveys can also cover aspects such as unreported crime, and obtain information on different levels with specified geographic areas and compare this with the reported crime. Such surveys have been available for more than 40 years and are now conducted in many countries to understand the local crime levels. Many conclusions have been drawn from victimisation surveys, for example, it was shown that more than half of the population said that they had been worried about being the victim of a crime sometime in the past Farrall et al. (1997); an increased fear of suffering a burglary was in fact correlated with the risk of suffering one Borooah and Carcach (1997); and minorities tend to be more fearful Brunton-Smith and Sturgis (2011).

The study of the fear of crime and the perception of security has many issues, which begin with the concept itself. Fear of crime and perception of security represent conceptually distinct constructs, although they have some similarities Wilcox Rountree and Land (1996), especially in the way both concepts have been studied Farrall et al. (1997). A person, for example, might perceive his or her own neighbourhood as being insecure, but at the same time might not be afraid of crime since the person does not consider themselves to be a potential target or considers that they have taken sufficient precautions to avoid being the victim, however, this is not the most common scenario.

This study relies on data from a victimisation survey conducted in Mexico INEGI (2014). It is based on the question “*In terms of crime, do you consider your region to be secure or insecure?*”, which is not exactly the same as the fear of crime. Although two different respondents might interpret the question differently, these differences should not be regional and so we can compare the answers provided by the survey respondents across Mexico. From here onwards we will use the term *perception of security* to refer to the answers provided in the surveys.

We develop a metric for the perception of security and then study its relationship with victimisation rates. The technique is not constrained to Mexico (or to surveys for that matter) and hence, the approach will have wider applications.

## A measure of the perception of security

To understand the perception of security attached to a particular region, we focus on the number of people surveyed that consider the region to be either secure or insecure, where the term *region* here might be as specific as a park or as general as a state. Typically in crime surveys, an individual might be asked whether a region is secure or not, so a binary answer is usually recorded. Other studies about the fear of crime or the perception of security have been conducted and the techniques used strongly depend on the type of data that is used. For example, the Crime Survey for England and Wales Office for National Statistics (2016) considers questions such as “*How safe do you feel walking alone in this area after dark?*”, and the respondent has four different options: 1-very safe, 2-fairly safe, 3-a bit unsafe, and 4-very unsafe; these responses provide an ordinal variable (where the order matters) and, in order to combine different questions and answers into a single number, a common technique is to assign a number to each response and sum them into a single *fear rate* Kershaw and Tseloni (2005), which might then be used in a statistical model Tseloni (2007).

Expressing the number of people who consider a region to be insecure as a ratio to the total number of people, produces a number between 0 and 1, where a value close to 0 means that the region is considered to be secure, and a value of close to 1 means that the region is considered to be insecure. Dividing our space (urban or otherwise) into a number of non-overlapping regions $R_1, R_2, \dots , R_n$, allows us to denote the perception of security of the general region $R_k$ by this ratio, which we denote as $s_k$. Thus, $s_k$ represents the mean perception of the surveyed population from a region and it provides an estimate of the probability that if we randomly select a person from the region $R_k$, then he or she considers that region to be insecure and, if enough surveys were conducted in the region $R_k$, then that number $s_k$ is a good estimate of that probability.

This is a simple way to quantify the perception of security of a region, however, this number $s_k$, by itself, is not particularly useful in isolation, since we do not know what the norm is, so for example, if a region gives us $s_k = 0.4$, is that considered to be high or low perception of security? It is much better to compare this number against the equivalent estimates in other regions or compare this regional measure over time to see the effect of either changing crime numbers or changing police strategies.

The fact that we can compare the perception of security between two different regions is the crucial point since it allows us to quantify the perception in a mathematical way which we may then use in a modelling context.

Given a method to establish the perception of security allows us to then rank all regions in our space. Assume for the moment that the perception of security from each region is different, and let $S_k \in \left\{ R_1, R_2, \dots R_n \right\}$ be the unique region which occupies the *k*-th position on the ranking of the perception of security, so $S_k$ reflects the perception of security of that region when compared to the other $n-1$ regions. The region $R_j$ such that $S_1 = R_j$ is considered the most insecure, since it occupies the first place in the ranking, and the region $R_l$ such that $S_n = R_l$ is considered the most secure, and is positioned in the last place of the ranking. Let $S = (S_1, S_2, \dots S_n)$ be the ranking obtained from the security perception, starting from the one considered to be the least secure up to the one considered the most secure. Now, if the survey considers a different type of question (or questions) to determine the perception of security from the survey respondent, it is also possible to either transform the response into a binary variable or to assign a number based on the order of the response Kershaw and Tseloni (2005). By either recoding the response or by considering the sum, we may obtain a similar ranking of the perception of security *S*.

We can now compare the perception of security between two different regions and state that, if in the ranking *S*, the region $R_k$ is listed before $R_j$ then this means that the region $R_k$ is considered to be less secure than $R_j$, so $s_k > s_j$. Thus, if we are considering *n* regions, then there are $n(n-1)/2$ comparisons by taking each pair of regions and selecting which of the two is considered to be the most secure and which the least.

**Fig. 1:**
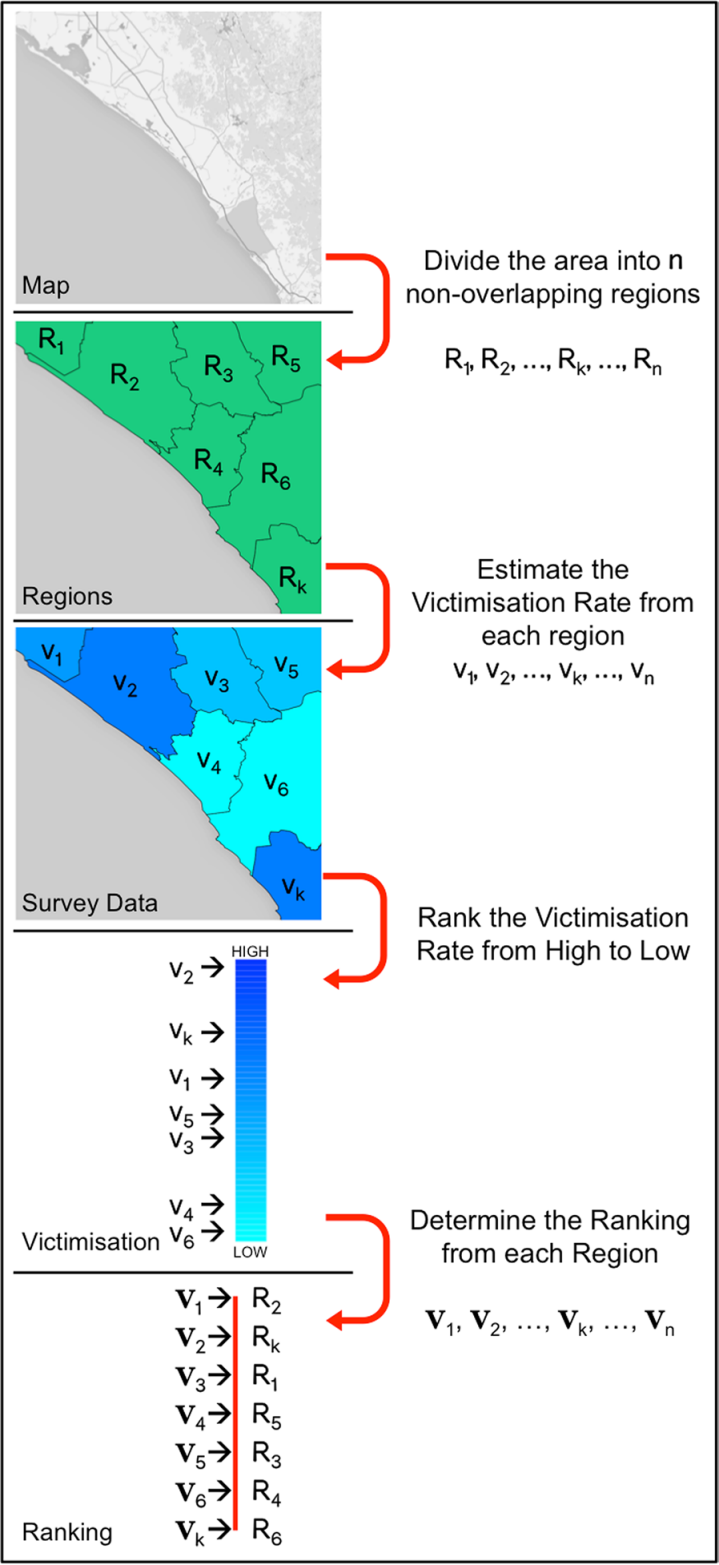
Process of dividing the area into non-overlapping regions, obtain their victimisation rate from survey data and rank the regions from the one with the highest victimisation rate to the one with the lowest victimisation rate

Suppose that now we can express the rate in which the region $R_k$ is victimised as $v_k$. This has many interpretations and depends on the type of crime and the time period considered, but let us suppose that $v_k$ represents the probability that a person suffers a particular type of crime, such as Robbery of a Person or Burglary, at least once, in a yearly period. Then $v_k$ provides information to compare two different regions and if $v_k > v_j$ it means that the population in the region $R_k$ suffers a higher probability of being the victim of a crime than the region $R_j$ for the type of crime considered. If we assume that the victimisation rate is different in every region, then it also provides a unique way to rank the *n* regions. Let $V_k \in \left\{ R_1, R_2, \dots R_n \right\}$ be the unique region which occupies the *k*-th position on the ranking of the victimisation rate. The region $R_j$ such that $V_1 = R_j$ has the highest victimisation rate and the region $R_l$ such that $V_n = R_l$ has the smallest victimisation rate. Let $V = (V_1, V_2, \dots , V_n)$ be the ranking obtained from that victimisation rate, as an ordered list of the *n* regions, from the one with the highest victimisation to the one with the lowest victimisation rates. The process of dividing into non-overlapping regions, taking into account the victimisation rate from each region and then ranking these victimisation rates is schematically drawn in Fig. 1; the process is the same for the perception of security ranking.

We now consider the victimisation rate and the perception of security as the ranking obtained when the regions are sorted from the one with the highest victimisation to the one with the lowest victimisation rate as *V* and from the one perceived as less secure to the one perceived as the most secure, as *S*. Our objective is to analyse the relationship between both rankings, so we need to consider all possible scenarios. For example, in the case of a tie, that is, if there are two regions such that $s_k = s_j$, then the ranking of the perception of security would not be unique, and a similar situation happens if there are two regions such that $v_k = v_j$. Since we are dealing with the mean perception of security and victimisation rates, ties are very unlikely to occur, but in order to avoid any inconsistencies, in the case in which $s_k = s_j$, then we use the index of the regions *j* and *k*, and if $j < k$ then the $R_j$ appears first in the ranking, and the same criteria is applied to the victimisation rate. The result is a unique ranking of the regions based on the perception of security and a unique ranking of the regions based on the victimisation rate.

From the perception of security and from the victimisation rate, we obtain two rankings, the perception of security *S* and the victimisation rate *V*, which may or may not be associated with each other and our objective is to quantify the degree of association. If the list consists of *n* elements, i.e., we are considering *n* different regions, then there is a total of $n(n-1)/2$ possible permutations, or distinct rankings in which the regions might be ordered. To determine the degree of similarity between the two rankings we consider a metric based on how far away is one ranking from the other, that is, how many movements would it take to go from one ranking to the other. More formally, we define a *swap* to be the permutation of any two neighbouring elements on the list, and a metric to compare *S* and *V* can be constructed by counting the minimal number of swaps required to go from one ranking to the other. For example, if the two rankings are identical, then it means that no swap is needed; if only the first two elements of *S* and *V* are in reversed order, then only one swap is needed and so on. The maximum number of swaps would occur in the scenario where *S* and *V* provide the same order, but reversed, so that the first place of *S* is the last place of *V* and so on, and in that case we would require $n(n-1)/2$ swaps to go from one ranking to the other. If we define *p* as the minimal number of swaps required to go from ranking *S* to ranking *V*, then we then define the *Ranking Metric* *P*(*S*, *V*) as

1 $$\begin{aligned} P(S,V) = \frac{n(n-1) - 4p}{n(n-1)}, \end{aligned}$$

which measures the number of swaps required to go from *S* into *V* and compares it against the maximum number of swaps. Since $p \in \left\{ 0, 1, \dots , n(n-1)/2 \right\}$, then $P(S,V) \in \left[ -1, 1\right]$. Thus, when $p = 0$ then $P(S,V) = 1$ and it means that rankings *S* and *V* are identical; a small value of *p* means that it only requires a few swaps to go from one ranking to the other, and we get a value of *P*(*S*, *V*) close to one. When *p* is closer to $n(n-1)/2$ it means that it requires most of the possible permutations, so *S* and *V* also have a relationship, only they provide a reversed order, and in this case, *P*(*S*, *V*) is closer to $-1$. Finally, when the number of swaps required to go from one ranking to the other is closer to $p = n(n-1)/4$, which is the middle between the largest and the smallest amount of swaps, then *P*(*S*, *V*) is closer to 0.

A similar problem arises when trying to compare the ranking provided by two different search engines Kumar and Vassilvitskii (2010). This metric is also known as Kendall Tau Rank Distance Shieh (1998) or the Kendall Rank Correlation Coefficient.

An alternative way to interpret such a metric is based on the multiple comparisons which the rankings *S* and *V* allow. If we select a pair of regions, $R_k$ and $R_j$ and compare their position in both *S* and *V*, then there are two possible scenarios, either both regions have the same order in both rankings, which means that in that comparison, the region with the highest victimisation rate is perceived as being less secure. The second scenario is that they do not preserve the same order, meaning that the region with more victims is considered to be more secure. The metric is defined as the number of times that a comparison preserves order in both of the rankings, *S* and *V*, against the total amount of comparisons that we can make by taking two different regions.

## Data description

We use the national victimisation survey conducted in Mexico in four yearly periods from 2011 to 2014, INEGI (2011, 2012, 2013, 2014) in which they ask, amongst others, the following questions:

- In terms of crime, do you consider your locality to be secure or insecure?
- In terms of crime, do you consider your county to be secure or insecure?
- In terms of crime, do you consider your state to be secure or insecure?

These questions help us understand the perception of security from people at three different geographic levels: locality, county and state, where the county is formed by a set of localities and a state is formed by a group of counties. The answers are binary, so the person being interviewed was only allowed to answer if he or she considered the region to be either secure or insecure in terms of crime.

Familiarity with the area reduces the worry about suffering a crime Gilchrist et al. (1998) which implies that smaller regions have a tendency of being perceived as more secure than larger regions, and so the state level is too general and gives non-comparable observations, if we take in account that there are some states (such as Chihuahua) which are larger in area than England, but there are some states (such as Morelos) which are smaller in area than Cyprus. Hence, we dismiss the measurements at a state level. The level of locality is, on the other hand, perhaps too specific and it does not provide a clear distinction in largely populated areas (such as Mexico City, which is divided into a few thousand localities). Hence, the region of measure of a county will be used in this study.

The way in which the survey was conducted considers the number of people from the population represented by each of the respondents based on their demographics so that the data contains an expansion factor, which is interpreted as the number of people represented by each observation and it goes between a few dozen to a few thousand. To avoid considering observations with only a small amount of respondents to the surveys, only counties with more than 300 people answering the survey in 2014 are considered, resulting in a total of 53 counties. Summary statistics for the perception of security data obtained for the 4 years considered is displayed in Table 1.

**Table 1:** Number of surveys considered and summary statistics for the perception of security, where a value closer to 0 means that in terms of crime people feel more secure and a value closer to 1 means that people tend to feel less secure

Year	Surveys considered	Perception of security			
		Mean	Min	Max	Std. deviation
2011	32,402	0.649	0.209	0.947	0.190
2012	42,033	0.631	0.169	0.932	0.194
2013	42,527	0.656	0.270	0.903	0.160
2014	34,544	0.660	0.246	0.922	0.153

**Fig. 2:**
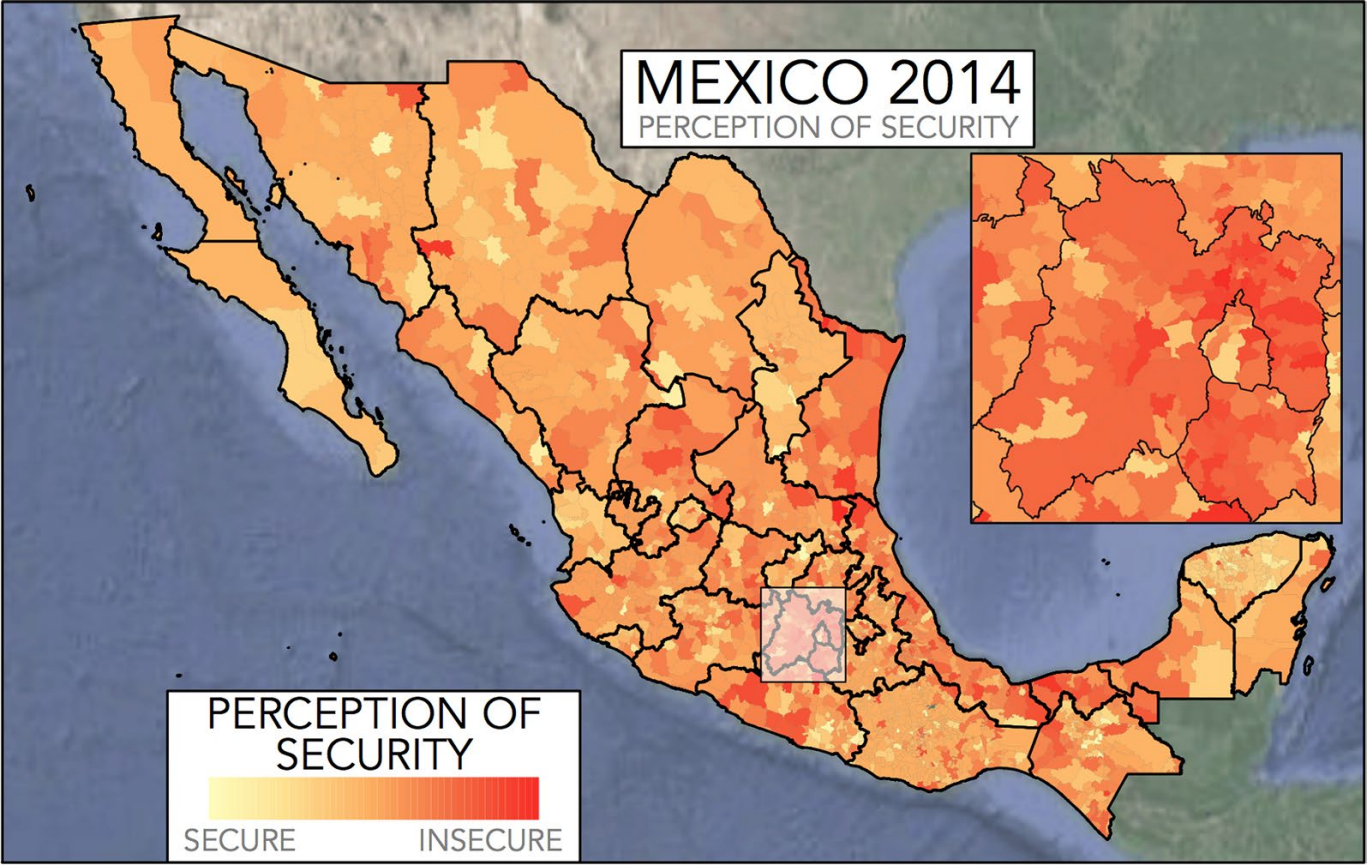
The perception of security in Mexico by counties. For counties with no information or small amount of survey respondents, the average perception of security, obtained as the average of the whole state is plotted, only for display purposes

The perception of security is a metric which allows us to differentiate counties based on the responses obtained from the survey. It shows, for example, that within the 4 years and in the 53 counties considered, the perception of security went as high as 0.947 (Ciudad Juárez in 2011), which can be interpreted as the probability that taking a person randomly from that county, he or she considered it to be insecure, with the answer being surprisingly high. The lowest perception of security (which means that the majority of the county considered it to be secure) was 0.169 (Mérida in 2012), which is a region of the country considered traditionally as being the most secure. This perception of security works as a quantitative measurement for the way in which a county is perceived and is not based on a single individual, so it gives a robust estimate for the regional perception of security and so it helps differentiate counties which are generally perceived as being secure to those counties which tend to be perceived as insecure. Figure 2 represents the perception of security from each county in Mexico during 2014. The perception of security is reasonably homogeneous, in that a county which is perceived as secure has similarly perceived counties as neighbours.

### Victimisation rates

Our interest is to relate the obtained metric for the perception of security to the victimisation rates. Here we measure the Ranking Metric *P*(*S*, *V*) based on the estimated perception of security and its relationship with self-reported crime. Eight different types of crime are considered:

1. Robbery of a Person
2. Car Theft (CT)
3. Partial Car Theft
4. Burglary
5. Vandalism
6. Kidnap
7. Murder
8. Missing Person.

The same survey that was used to construct the measure of the perception of security is used to estimate the victimisation rates. From the same survey, they ask, amongst others, the following questions:

- During the previous year, did any member of this home, including yourself, suffered a *type of crime being considered*?
- How many times did you or any member of this home suffered a *type of crime being considered* during the previous year?

These two questions are asked for each type of crime and help us understand the different victimisation rates in each of the regions. In the case of Robbery of a Person the question does not include other members of the home, and in the case or Murder, Missing Person and Kidnap the question is asked only with regards to the members of the home. All cases are self-reported, so it depends on the ability of the survey respondents to recall their experiences in terms of crime Farrall et al. (1997) and so there may be sources of error, but nevertheless they lead to valuable information. This part could be computed with police recorded crime data, however, in Mexico, less than 8% of the crimes gets recorded by the police INEGI (2014).

**Fig. 3:**
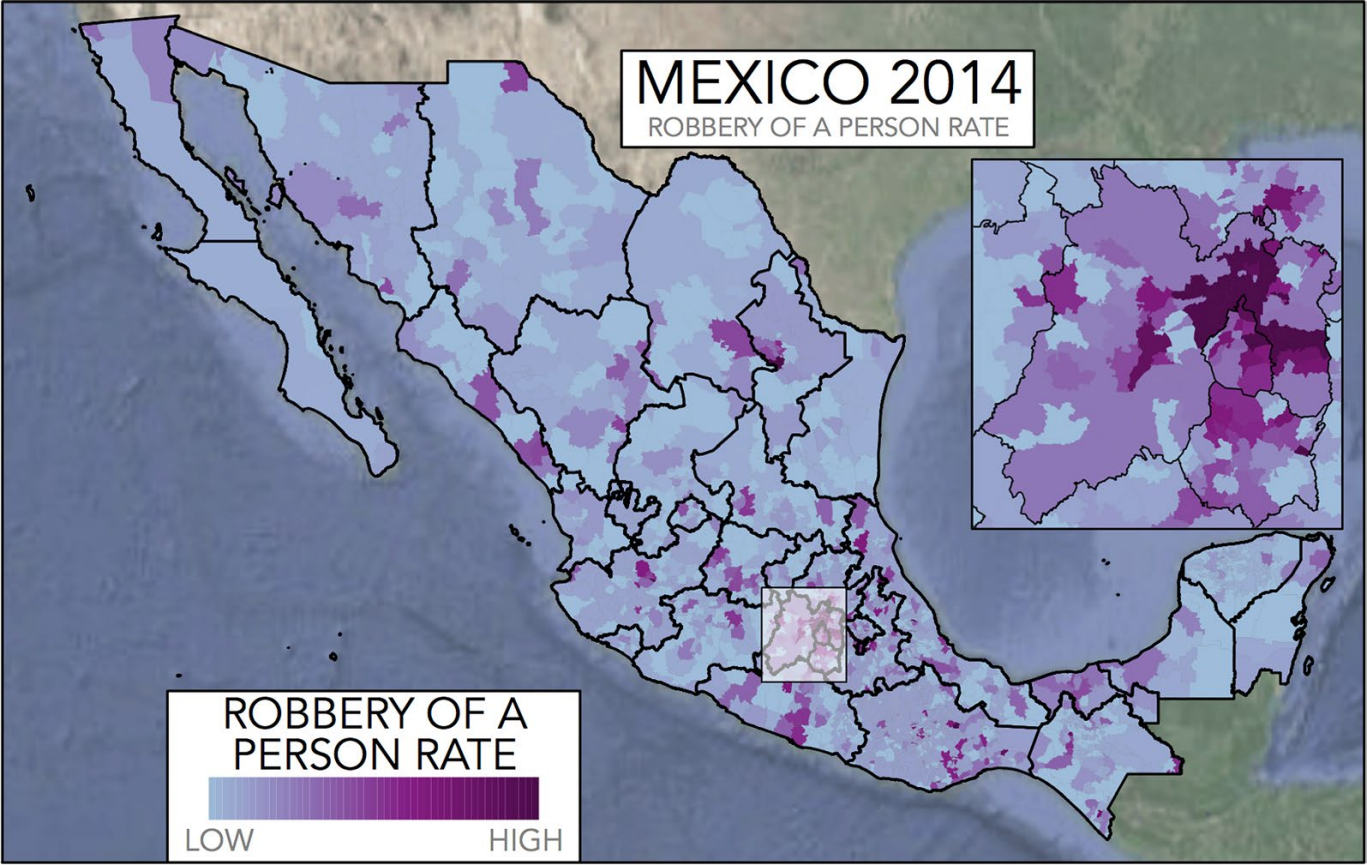
The victimisation rate in Mexico considering Robbery of a Person in 2014. For counties with no information or small amount of survey respondents, the average victimisation rate, obtained as the average of the whole state is plotted, only for display purposes

The victimisation rates are obtained from the survey, and so for each county we obtain an estimate of the number of victims of each type of crime during a year, expressed as $v^{(i)}_k$ for the *i*-th type of crime and for the *k*-th county. Reported victimisation rates for the eight types of crime considered are reported in Table 2 and the victimisation rates for the case of Robbery of a Person, $v^{(1)}_k$, are displayed in Fig. 3.

**Table 2:** Victimisation rates, range and standard deviation for the eight types of crime considered in Mexico during 2014 (data obtained from the victimisation survey)

Crime	Mean	Min	Max	Std. deviation
Robbery of a person $v^{(1)}$	0.091	0.007	0.45	0.066
Car Theft (CT) $v^{(2)}$	0.030	0.002	0.096	0.019
Partial CT $v^{(3)}$	0.125	0.025	0.250	0.054
Burglary $v^{(4)}$	0.066	0.023	0.149	0.025
Vandalism $v^{(5)}$	0.107	0.014	0.222	0.053
Kidnap $v^{(6)}$	0.004	0	0.014	0.003
Murder $v^{(7)}$	0.001	0	0.007	0.001
Missing person $v^{(8)}$	0.002	0	0.006	0.001

## Perception of security and victimisation rates

**Fig. 4:**
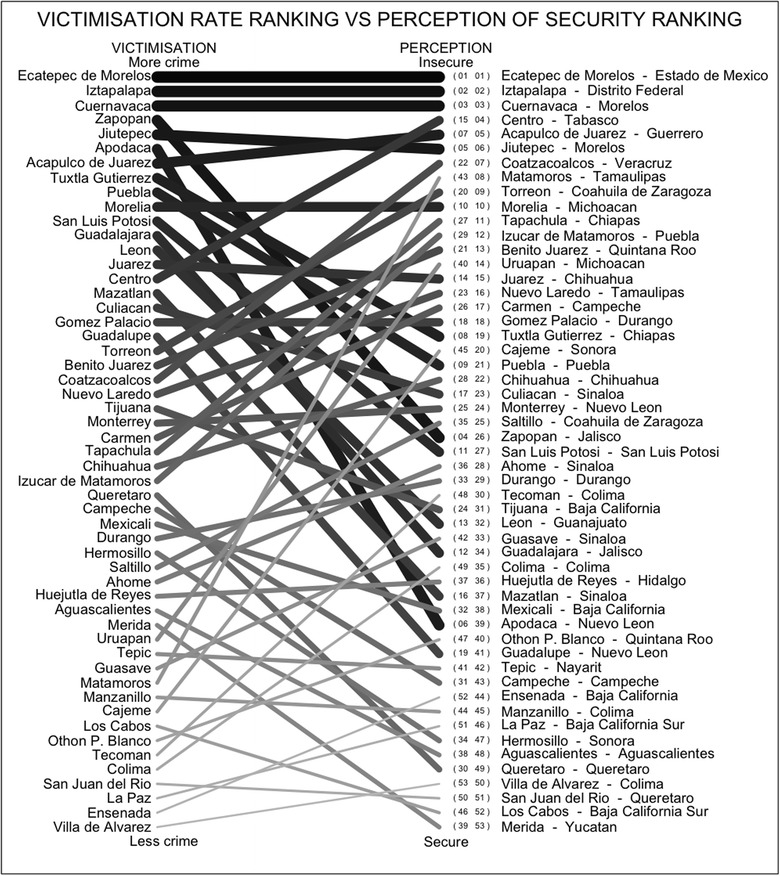
The ordering in the *first column* shows the victimisation rate ranking of Robbery of a Person obtained from the 53 counties in Mexico and in the *second column* is the ranking of the perception of security/insecurity from that county. Places with a low level of victimisation are usually ranked as secure. The *numbers* inside the *brackets* (in *small font*) are the actual rankings from each on the regions on both of the lists, that is *V* and *S*

Robbery of a Person is used as an initial approach to the victimisation rates hence $V^{(1)}$ is the ranking from the 53 counties, from the one which suffers the highest amount of robbery of a person, to the one which suffers the lowest. The Ranking Metric based on the permutations is displayed in Fig. 4, where the first column is the victimisation rate ranking, $V^{(1)}$, with the counties that suffer the higher rates in the upper part, and the second column displays the perception of security ranking, *S*, with the counties perceived as the least secure in the upper part. A line is drawn between the same county in the two rankings. Hence, a horizontal line indicates that a county is ranked in the same place in both $V^{(1)}$ and *S*, and intersections between any two lines means that the corresponding counties are not in the same order in both rankings. Perhaps as expected, in Fig. 4 we can identify that it is not usual for a county to have a high (low) victimisation rate and to be identified as secure (insecure).

**Fig. 5:**
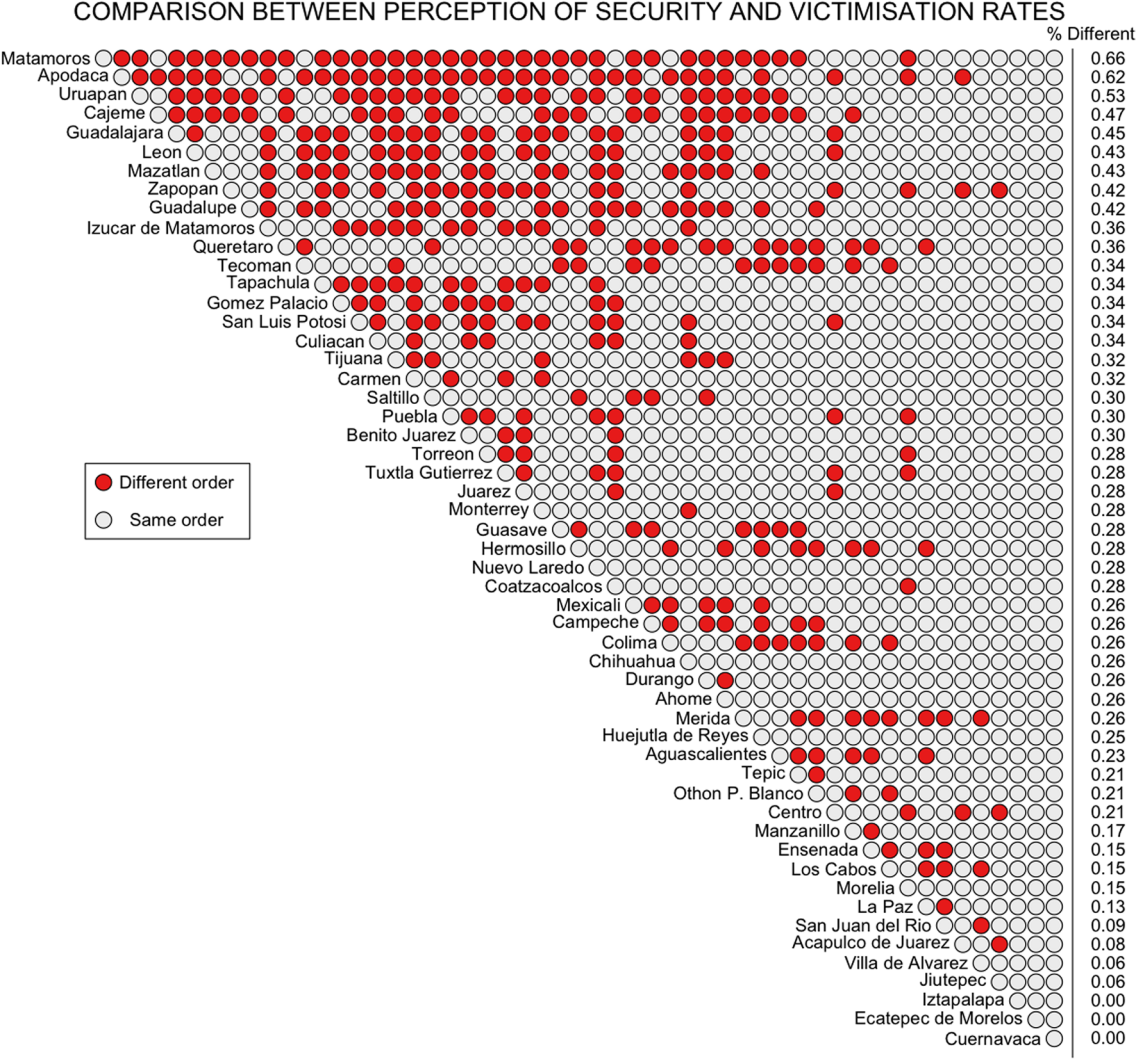
Displayed are the 1653 comparisons that were made from the selected 53 counties in Mexico, using a *red mark* to highlight a comparison that does not preserve order, meaning that places with higher victimisation of Robbery of a Person are nonetheless considered more secure

Using the same data, both rankings $V^{(1)}$ and *S* might be displayed as an upper triangular matrix $T_{ij}$, with each of the counties as the rows and columns of $T_{ij}$ and the result of the comparison between the *i*-th and the *j*-th county as the entry (*i*, *j*), where we assign a value of 1 if these two counties have a different order in both rankings and a value of 0 otherwise (so that $T_{ij}$ helps us identify every pair of counties in which the one with the smaller victimisation rates is perceived as being less secure). If we sum all the entries of $T_{ij}$ we obtain *p*, the number of swaps required to go from one of the rankings to the other. Using different colours to identify the entries of $T_{ij}$ which are either 1 or 0, the results of the comparison between each pair of counties is displayed in Fig. 5. The column on the right-hand side displays the percentage of counties which have a different order against the corresponding county, and it reveals that the counties with the lowest number of differences are those in which their victimisation level is either so high or so low that they are easily identifiable as secure or insecure. However, the counties which have a higher number of differences are the in which the victimisation does not correspond to the perception of security. Two examples are the counties of Apodaca and Guadalupe, both in the Nuevo León state, which have a high victimisation level but are perceived as relatively secure.

When investigating the relationship obtained between the perception of security and the victimisation rate, the value of the metric and its display might, statistically speaking, be the result of randomness. However, there is strong evidence to counteract this argument. We can consider a Null Hypothesis of no correlation between the rankings *S* and $V^{(1)}$, in which the metric $P(S,V^{(1)})$ has an expected value of zero. For a very small number of elements in the rankings, it is possible to compute the exact distribution and obtain a confidence interval for the expected value of $P(S,V^{(1)})$ under that hypothesis, whilst for a large amount of elements, the variance can be approximated Kendall (1948) by

2 $$\begin{aligned} Var(P) = \frac{2(2n+5)}{9n(n-1)}. \end{aligned}$$

**Fig. 6:**
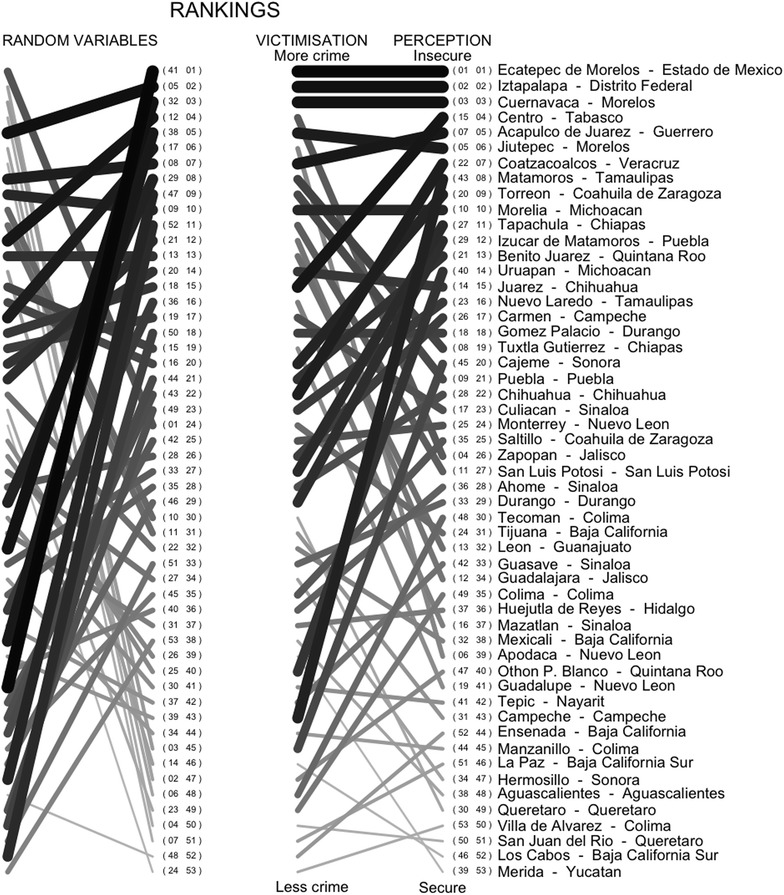
Comparison of random variables with no relationship between each pairing on the *left-hand side*, and the relationship between Robbery of a Person and perception of security obtained through victimisation surveys, on the *right-hand side*. As observed, random rankings create a more tangled pattern

However, we are considering 53 counties here, which is not small enough to compute the exact distribution, nor is it large enough to trust the approximated variance. Instead, if we simulate two random variables with a length of 53 cases each and we focus on both rankings, the result is not usually an ordered structure, compared to the one observed in Fig. 4, but is more similar to a completely disordered graph, with tangled lines, as the one displayed on the left-hand side of Fig. 6.

**Fig. 7:**
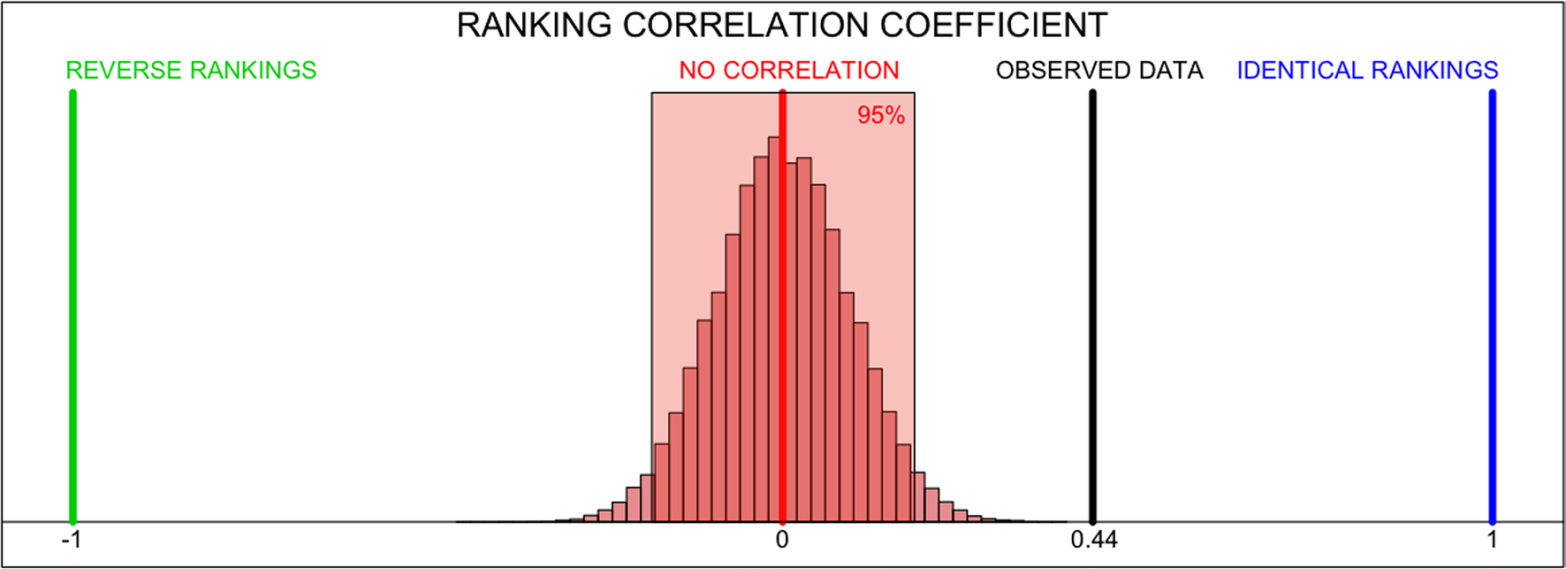
Histogram that shows the metric *P*(*S*, *V*) of 2000 simulated rankings and the value observed from data. The *red* area contains 95% of the simulations, obtained inside the $(-0.182, 0.182)$ interval. The observed value of 0.44 is far away from any simulated result, meaning that we can reject that the rankings *S* and *V* are independent

From the 53 different counties, the value of the $P(S,V^{(1)})$ is 0.44, as displayed in Fig. 4 but, is that value enough to reject the Null Hypothesis that the perception of security ranking and the victimisation rate ranking are not related? A simulation of 2000 random rankings is useful to convince us that these variables are indeed related, as displayed in Fig. 7. From the 2000 random rankings, 95% were found to have a Ranking Metric that lies between −0.182 and 0.182, where any value above that interval, as in the observations from the counties in Mexico, is interpreted as a clear relationship between the two rankings. Observed values below the (−0.182, 0.182) interval obtained through the simulation are also considered as a clear relationship between the rankings, only having a reversed order, which is what we would obtain if instead of ranking counties from the one with the most victimisation we started from that one with the least victimisation. The interval obtained through the simulations is very close to the (−0.185, 0.185) which we would have obtained if we used the approximated value of the variance, from Eq. 2.

The Ranking Metric might also be used to track the changes of the perception of security over different time periods. If we let $S^{2013}$ be the perception of security ranking for the year 2013 and $S^{2014}$ for 2014, then we can measure $P(S^{2013}, S^{2014})$ which tells us how the Ranking Metric of the perception of security compares for the two consecutive years, and similarly if we take the Ranking Metric between two victimisation rate rankings. Results for the Ranking Metric between the perception of security and victimisation rates for different years is displayed in Table 3.

**Table 3:** Ranking Metric between the perception of security on the left, and the victimisation rates on the right, between 2011 and 2014

	Perception of security ranking *S*			Victimisation ranking *V*		
	2012	2013	2014	2012	2013	2014
2011	0.6286	0.5604	0.5240	0.6604	0.5806	0.6546
2012	–	0.5720	0.5008	–	0.5864	0.6430
2013	–	–	0.6996	–	–	0.6648

A value closer to 1 means that the rankings have a higher degree of correlation, a value closer to 0 means no correlation between the rankings, and a value closer to −1 means that the rankings provide a reverse order

The perception of security ranking tends to be more closely related between any two consecutive years, as expressed in Table 3, but this is not always the case since for example, the $S^{2014}$ ranking is more similar to the $S^{2011}$ than it is to the $S^{2012}$. This shows that in general the perception of security is set for a considerably large amount of time, and so a region that in the past was perceived as being secure (insecure) has a tendency of being perceived secure (insecure) in subsequent years. There is, after all, a memory in the system.

The victimisation rankings follow a different pattern, for example, the ranking of the year 2011 is more similar to the 2014 ranking than to the 2013. This shows that there are some counties with low (high) victimisation rate in 2011 which had a higher (lower) victimisation rate for the year 2013 but then went back in the year 2014.

**Fig. 8:**
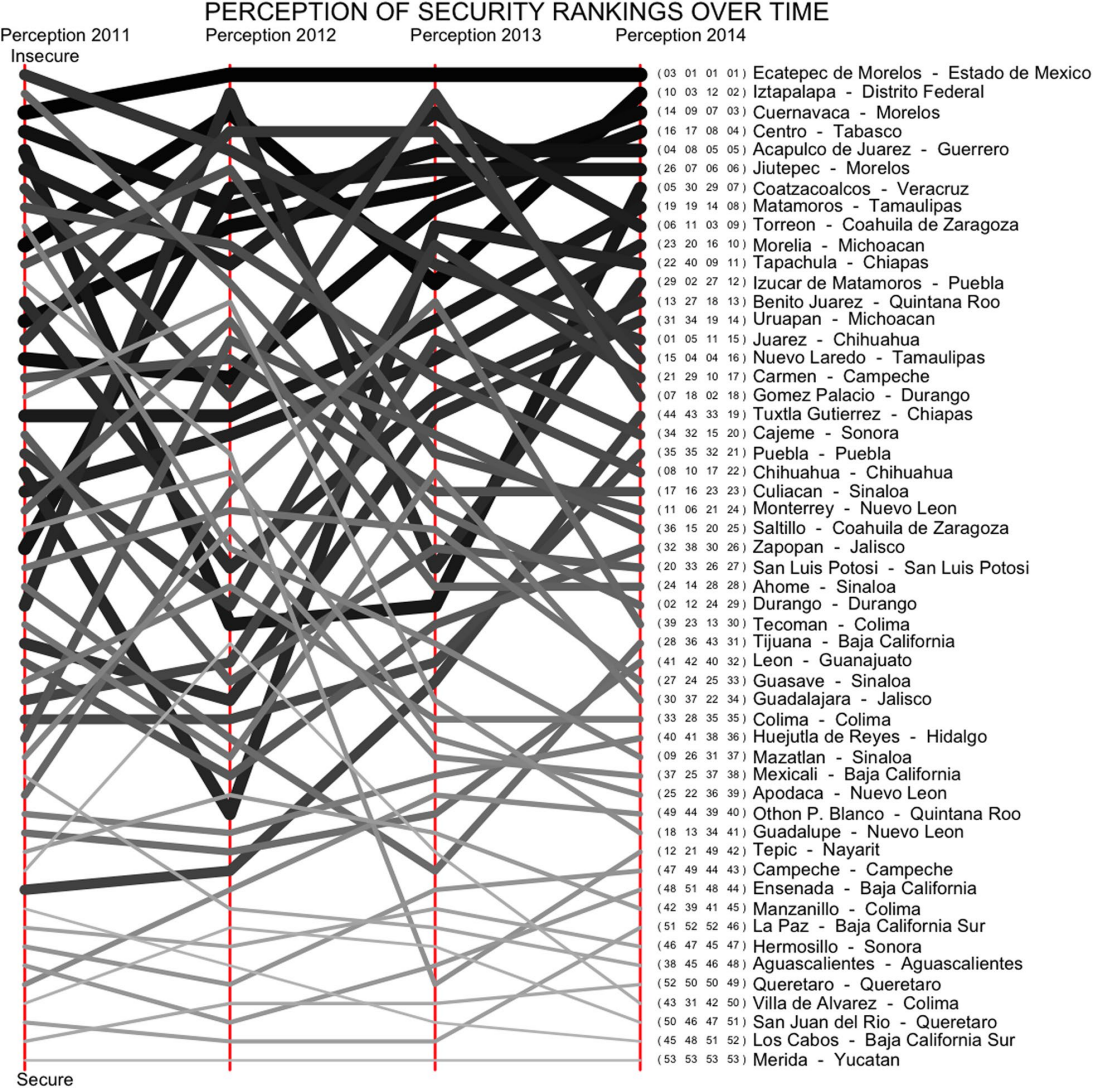
The perception of security ranking *S* of the 53 counties being considered in Mexico from 2011 to 2014. In *brackets* is the four consecutive rankings from that region. It shows that it is common for a region to have similar rankings in consecutive years

The perception of security ranking has a small degree of variability between consecutive years, as displayed in Fig. 8, particularly for the counties which are considered secure. Two relevant and interesting cases are Ciudad Juárez and Chihuahua, both located in the state of Chihuahua, which occupied the 1st and 8th place as the most insecure counties in 2011 and they are located in the 2014 survey in the 15th and 22nd place respectively. The perception of security from these two counties has improved considerably as compared to the rest of the counties considered.

## Results

The Ranking Metric $P(S, V^{(i)})$ can be computed using data for different types of crime and, since the ranking obtained by sorting the counties based on the victimisation rate of Robbery of a Person is different to the ranking of the other types of crime, we obtain different results for the $P(S, V^{(i)})$ metric. We compare the Ranking Metric $P(S, V^{(i)})$ obtained by ranking the different types of crime alone and the results are displayed in Table 4.

**Table 4:** Ranking Metric between the perception of security and the different types of crime

Crime	Ranking Metric
	$$P(S, V^{(i)})$$
Robbery of a person	0.4368^a^
Car Theft (CT)	0.1292
Partial CT	−0.0522
Burglary	0.0538
Vandalism	−0.0290
Kidnap	0.2510^a^
Murder	0.1002
Missing person	0.1902^a^

A value closer to 1 means that the rankings have a higher degree of correlation, and a value closer to 0 means no correlation

^a^Values that are statistically different to zero

At first glance, the results in Table 4 show that the ranking of most of the types of crime is statistically not related to the perception of security ranking, since again, most of them lie between the $(-0.18, 0.18)$ rejection interval obtained through simulation. This means that, for example, if we rank the 53 counties which we consider here based on their Murder rates (from the ones with the highest rate to the ones with the smallest rate) and we compare that ranking to the perception of security ranking, they would display no relation. This result might seem surprising, but is a reflection that murder is a rare event, so the counties usually have a rate so close to zero that the murder ranking becomes almost irrelevant.

It is relevant to note that there are some counties, such as Uruapan and Matamoros, in which the perception of security does not match the victimisation rates, and they tend to be the ones with the highest number of comparisons with different order between the victimisation ranking and the perception of security ranking, as displayed in Fig. 5. This means that these counties may have particular situations, perhaps, such as organised crime, or a high victimisation background.

There are, on the other hand, counties such as Apodaca and Guadalupe, both in the state of Nuevo León, as well as Guadalajara and Zapopan in the state of Jalisco, as well as Mazatlán in the state of Sinaloa, which are perceived as being much more secure than we would perhaps expect, based on their victimisation rates. These four counties were perceived as much less secure a few years ago, as displayed in Fig. 8, particularly Mazatlán, which was in the 10th place as the least secure county in 2011 but in 2014 occupies the place 37th. It is possible that these highlighted cases might be counties where organised crime and other types of crime such as extortion have had an impact on the society and its perception of security, without crime itself being reflected in the eight victimisation rates considered in the model.

## Conclusions and discussion

In this paper, the methodology presented constructs a regional metric for the perception of security which can be easily interpreted as the probability that a person perceives that region to be insecure. Even when the perception of security is based on the impressions and fears from a surveyed population, with these feelings representing a generalised behaviour in the population, the perception of security might be considered an attribute of the region rather than the impressions and fears of some of its individuals, hence the validity of a regional approach to the perception of security.

Quantitatively speaking, although the perception of security by itself does reveal a pattern of beliefs, the absolute measure is much more valuable when compared to other regions or over different time periods since the significance of the isolated number cannot be easily determined. This regional perception of security allows us to compare and differentiate two regions based on their perceptions. Ranking the perception of security gives a valuable insight of the feelings of the population in terms of crime and it allows us to compare many regions simultaneously. This approach is not constrained to Mexico or to the geographic level of counties and thus more specific regions could also be ranked by their perception of security.

Due to many reasons (such as the lack of information, the fact that crime is a rare event and the important role that the media plays) a person might not be able to correctly predict their own chances of suffering a particular type of crime. The actual chance of being the victim of a crime may differ highly from how that person perceives insecurity where he or she lives. If a region is generally deemed as being insecure, regardless of its victimisation rates, then its population reacts to that perception. A high level in the perception of insecurity may cause people to change their behaviour, for instance by changing their shopping or eating trips to the neighbourhoods which are perceived to be insecure. In Ciudad Juárez, for example, 92% of the population considered their county to be insecure in 2011 and as a result of that climate of insecurity, some people actually moved cities when their small businesses became less profitable, half of them crossing the border into the United States Albuja, (2014). Between 2006 and 2011 nearly 1.7 million people were internally displaced in Mexico because of their perceived insecurity and the violent atmosphere Cantor (2014). The extreme perception of insecurity becomes as relevant a concern as crime itself.

The perception of security is affected by past circumstances, which in terms of policy design shows that events, such as a kidnap or death of a child covered extensively in the media, for example, might have an immediate negative impact whose influence may extend for some time so that improvements in the perception of security tend to be quite slow. Therefore, even if a county is suddenly successful at reducing its victimisation rates, it might take a long time for the perception to recover. However, a decline in the perception of security might happen rapidly, as seen in the perceptions in Coatzacoalcos, Veracruz from the 30th and 29th place in 2012 and 2013 to the 7th place of insecurity in 2014, which clearly indicates a new concern in that county. In Coatzacoalcos, the perception of security went from 0.65 in 2013 to 0.80 in 2014 so that from 1 year to the next one, 15% of the population from that county changed their position and considering it to be insecure. Interestingly, between 2013 and 2014, most of the victimisation rates in Coatzacoalcos actually decreased but the number of kidnaps more than doubled and so, which might indicate that the increased perception of insecurity is the result of the increase in the kidnap rate.

Tracing the changes in the victimisation rates for different types of crime and for many counties is a multidimensional problem, but tracing any drastic changes in the perception of security, that is, simply ranking counties over consecutive years, may reveal the emergence of a concern about crime in a particular region. It may be much simpler to identify the reasons that cause it, as for the case of the increase of the kidnap rates in Coatzacoalcos. If the population feels more insecure, something needs to be done. The measure of perception can thus be used alongside other indicators to try and identify shifts in social norms.

The perception of security has a high Ranking Metric with Robbery of a Person, meaning that counties in which Robbery of a Person is more frequent tend to be perceived as being less secure. Robbery of a Person is the second most frequent type of crime in Mexico and it is a type of crime in which the victim and the criminal have some form of contact, at least for a few seconds, and perhaps not surprisingly, this type of crime then has the highest ranking correlation. The other two types of crime with a high Ranking Metric are Missing Person and Kidnap, but these two types of crime are relevant due to their high social impact rather than their frequency. In Mexico during 2014, for every 119 Robberies of a Person, there was a single Kidnap. The fact that Partial Car Theft and Vandalism have a low ranking correlation with the perception of security, taking into account that they are the first and third most frequent type of crime respectively, shows that low impact crime has, in fact, a low impact on the perception of security, especially if the population has more relevant (although perhaps much less frequent) crimes to worry about.

Results here highlight that efforts invested in reducing the levels of lower impact crimes (such as Vandalism or Partial Car Theft) might not actually improve the perception of security, even when they are the most frequent types of crime. However, a policy oriented to reduce the levels of Robbery a Person might have much better results in terms of its effect on the perception of security since it has a strong impact on the perception of security accompanied with a relatively high frequency.

This research revealed that Ecatepec, Estado de México, located in the metropolitan area of Mexico City, has been ranked as the most insecure county for the past 3 years. This is perhaps not surprising since between 2011 and 2014 the victimisation rates for Robbery of a Person and Car Theft have more than doubled. An intervention oriented to reduce its victimisation rates is clearly needed. It would then be interesting to also monitor its perception of security, particularly if this could be compared with a county where a different approach was taken.
